# The Influence of Angiotensin Peptides on Survival and Motility of Human High-Grade Serous Ovarian Cancer Cells in Serum Starvation Conditions

**DOI:** 10.3390/ijms23010052

**Published:** 2021-12-21

**Authors:** Kamila Domińska, Kinga Anna Urbanek, Karolina Kowalska, Dominika Ewa Habrowska-Górczyńska, Marta Justyna Kozieł, Tomasz Ochędalski, Agnieszka Wanda Piastowska-Ciesielska

**Affiliations:** 1 Department of Comparative Endocrinology, Medical University of Lodz, Zeligowskiego 7/9, 90-752 Lodz, Poland; tomasz.ochedalski@umed.lodz.pl; 2 Department of Cell Cultures and Genomic Analysis, Medical University of Lodz, Zeligowskiego 7/9, 90-752 Lodz, Poland; kinga.urbanek@umed.lodz.pl (K.A.U.); karolina.kowalska1@umed.lodz.pl (K.K.); dominika.habrowska@umed.lodz.pl (D.E.H.-G.); marta.koziel@umed.lodz.pl (M.J.K.); agnieszka.piastowska@umed.lodz.pl (A.W.P.-C.)

**Keywords:** Ang-(1-7), Ang-(1-9), Ang-(3-7), ovarian cancer, OVPA8, serum starvation, cell survival, cell migration

## Abstract

High-grade serous ovarian carcinoma (HGSOC) is the most frequent and malignant form of ovarian cancer. A local renin–angiotensin system (RAS) has been found in the ovary, and changes in selected components of this system were observed in pathological states and also in ovarian cancer. In the present study, we examined the effect of three peptides, Ang-(1-7), Ang-(1-9) and Ang-(3-7), on proliferation and motility of the OVPA8 cell line, a new well-defined and preclinical model of HGSOC. We confirmed the presence of mRNA for all angiotensin receptors in the tested cells. Furthermore, our findings indicate that all tested angiotensin peptides increased the metabolic serum in the medium by activation of cell defense mechanisms such as nuclear factor kappaB signaling pathway andapoptosis. Moreover, tested angiotensin peptides intensified serum starvation-induced cell cycle arrest at the G0/G1 phase. In the case of Ang-(3-7), a significant decrease in the number of Ki67 positive cells (Ki67+) and reduced percentage of activated ERK1/2 levels in ovarian cancer cells were additionally reported. The angiotensin-induced effect of the accumulation of cells in the G0/G1 phase was not observed in OVPA8 cells growing on the medium with 10% FBS. Moreover, in the case of Ang-(3-7), the tendency was quite the opposite. Ang-(1-7) but not Ang-(1-9) or Ang-(3-7) increased the mobility of reluctant-to-migrate OVAP8 cells cultured in the serum-free medium. In any cases, the changes in the expression of *VIM* and *HIF1A* gene, associated with epithelial–mesenchymal transition (EMT), were not observed. In conclusion, we speculate that the adaptation to starvation in nutrient-deprived tumors can be modulated by peptides from the renin–angiotensin system. The influence of angiotensin peptides on cancer cells is highly dependent on the availability of growth factors and nutrients.

## 1. Introduction

The changes in selected components of the local renin–angiotensin systems were observed in pathological states of the reproductive tissues, including the ovary. The literature confirms the involvement of angiotensin peptides and their receptors in the occurrence and development of ovarian diseases such as polycystic ovary syndrome, ovarian hyperstimulation syndrome and ovarian cancer [1]. Ovarian cancer (OC) accounts for only a small percent of all cancers in women but is one of the deadliest. OC is a heterogeneous disease comprising several histologic types. The most common form and the one with the worst prognosis is ovarian cancer, which originates mainly from the fallopian tube [2]. For these reasons, we decided to use the OVPA8 cell line that is a good high-grade serous ovarian carcinoma (HGSOC) model. This ovarian cancer line presents morphologic, genetic and functional features consistent with HGSOC [3].

The mechanisms linking RAS with carcinogenesis describe two opposing signaling pathways: ACE1/Ang II/AT1 axis increasing cellular divisions and cell migration and invasion and the ACE2/Ang-(1-7)/Mas axis, which inhibits the proliferation and metastasis potential of cancer cells [1,4,5,6]. However, there are many deviations from the classic scheme, as other active hormonal peptides of RAS, apart from Ang II and Ang-(1-7), or other receptors for angiotensin, apart from AT1 and MAS, are involved. Moreover, the pleiotropic character of angiotensins and their multidirectional, tissue-specific activity are also important. There are many questions still to be answered and numerous unexplained discrepancies that emphasize the need for further research. In the present study, we examined the effect of three peptides, Ang-(1-7), Ang-(1-9) and Ang-(3-7), on the proliferation and motility of high-grade serous ovarian cancer cells. All three angiotensin peptides belong to the bioactive peptides of the renin–angiotensin system. The experiments were carried out in a standard culture medium with 10% fetal bovine serum (FBS) as well as in a medium without FBS (serum starvation).

## 2. Results

### 2.1. The Changes in OVPA8 Cells Viability, Metabolic Activity and Proliferation after Angiotensin Peptides Exposure

**Figure 1:**
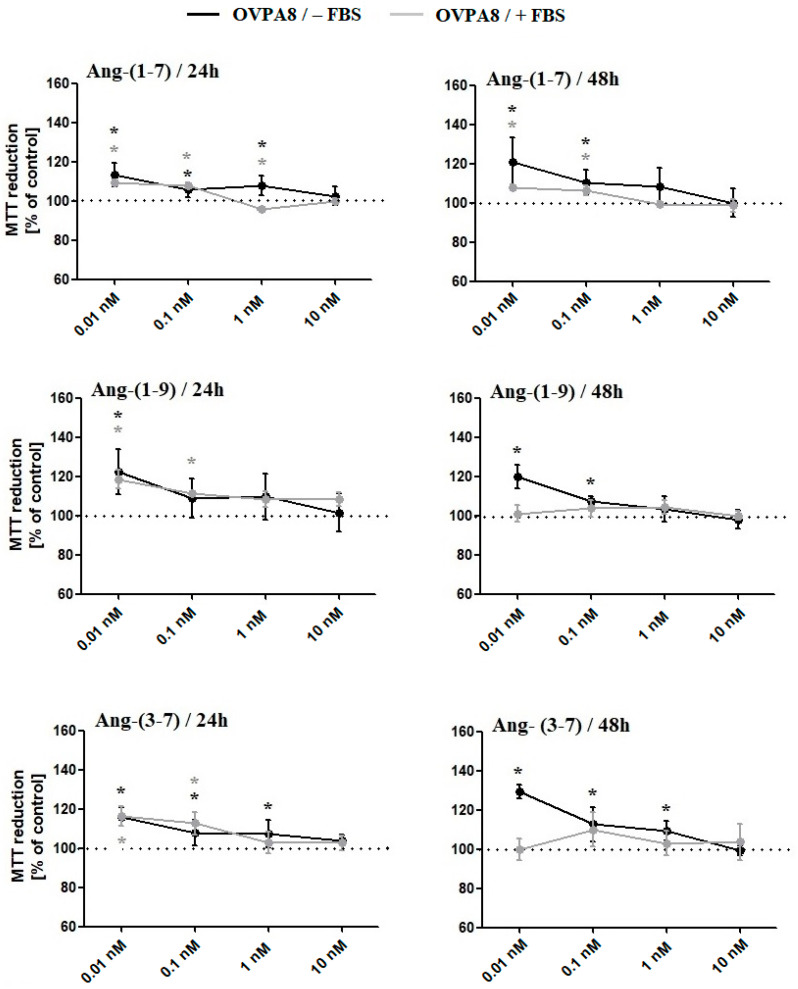
The MTT Assay results following the incubation of OVPA8 cells with tested angiotensins in the medium with or without FBS (mean ± SD; one-way ANOVA with Tukey’s test: * *p* < 0.05).

**Figure 2:**
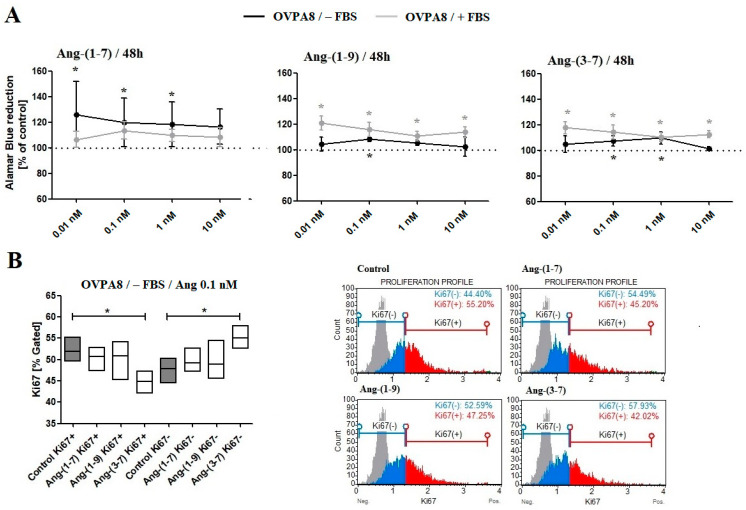
(**A**) The Alamar Blue Assay results, following the incubation (48 h) of ovarian cancer cells with tested angiotensins in the medium with or without FBS. (**B**) The Muse^®^ Ki67 Proliferation Assay results following the incubation (48 h) of ovarian cancer cells with 0.1 nM angiotensins in the medium without FBS (mean ± SD; one-way ANOVA with Tukey’s test: * *p* < 0.05).

MTT (Figure 1) and Alamar Blue (Figure 2A) Assays showed that all tested angiotensin peptides increased the metabolic activity of OVPA8 cells; however, the results were most often statistically significant for lower concentrations (0.01 and 0.1 nM). The presence or absence of fetal bovine serum in the medium did not affect the results for tetrazolium salt 3-(4,5-dimethylthiazol-2-yl)-2,5-diphenyl tetrazolium bromide (MTT) reduction (exception: Ang-(1-7)/1 nM/24 h; Ang-(1-9)/0.01 nM/48 h; Ang-(3-7)/0.01 nM/48 h) but it was visible in the case of resazurin reduction: Ang-(1-7)/0.01 nM; Ang-(1-9) and Ang-(3-7)/10 nM, 0.1 nM, 0.01 nM. The mRNA level of survivin (*BIRC5*), a member of the inhibitor of apoptosis family, was elevated for Ang-(1-7) and Ang-(1-9) but statistically was not significant in any cases. On the other hand, the ratio of *BCL2* to *BAX* expression levels significantly increased only for Ang-(3-7) (Table 1).

**Figure 3:**
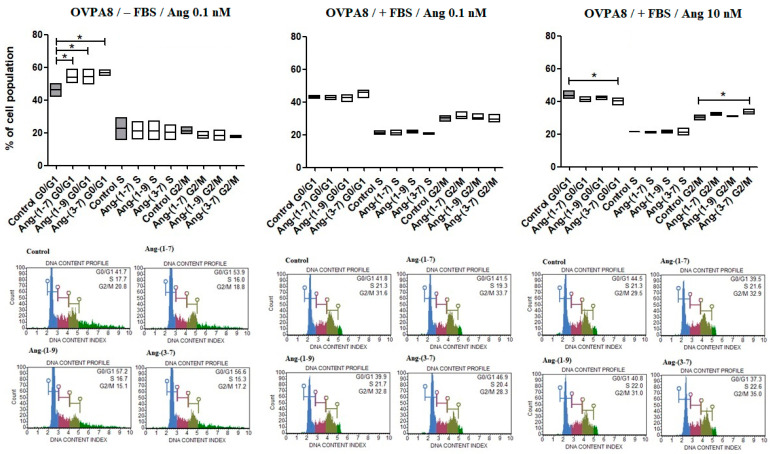
The Muse Cell Cycle Assay results following the incubation (48 h) of OVPA8 cells with tested angiotensins in the medium with or without FBS (mean ± SD; one-way ANOVA with Tukey’s test: * *p* < 0.05).

After 48 h incubation with tested peptides (0.1 nM), a substantial increase in cells in G0/G1 phase was observed, however, only in the medium without FBS. What is interesting is that angiotensins at the concentration of 10 nM, served in the medium with 10% FBS, decreased the number of cells in the G0/G1 phase but significantly only for Ang-(3-7). In the case of Ang-(3-7) (10 nM), we also observed increased cell population in the G2/M phase (Figure 3). Muse Ki67 Proliferation Assay showed a significant decrease in the number of Ki67 positive cells (Ki67+) after 48 h incubation with Ang-(3-7) (0.1 nM; the medium without FBS) (Figure 2B), but we did not observe decreased mRNA expression of *MKI67* after treating OVPA8 cells with any angiotensin (Table 1).

### 2.2. The Changes in OVPA8 Cell Mobility after Angiotensin Peptides Exposure

**Figure 4:**
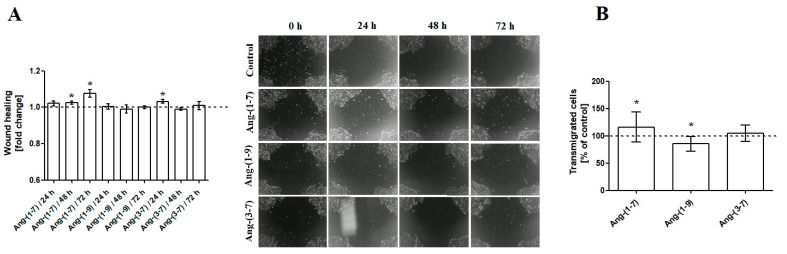
(**A**) The Wound Healing Assay and (**B**) Boyden Chamber Assay results, following the incubation (48 h) of OVPA8 cells with tested angiotensins in the medium without FBS (mean ± SD; one-way ANOVA with Tukey’s test: * *p* < 0.05).

As shown in Figure 4, Ang-(1-7) enhances the mobility of ovarian cancer cells in both the Wound Healing Assay (Figure 4A) and Transmigration Assay (Figure 4B). Ang-(1-9) does not facilitate colonization of OVPA8 cells into new areas but decreases cell migration through a PET membrane with the 8 μm pore. Ang-(3-7) initially increases the rate of migration (24 h); however, with a longer period of time (48 h and 72 h), the effect is no longer visible (Figure 4). Gene expression analyses revealed no marked change in the *VIM* gene expression. The incubation of OVPA8 cells with Ang-(1-9) and Ang-(3-7) resulted in a slight decrease in the expression. The mRNA level of hypoxia-inducible factor 1 (*HIF1A*), which promotes migration and extracellular matrix remodeling, also remained unchanged (Table 1).

### 2.3. The Changes in MAPK Phosphorylation Relative to the Total MAPK Expression in OVPA8 Cells after Angiotensin Peptides Exposure

**Figure 5:**
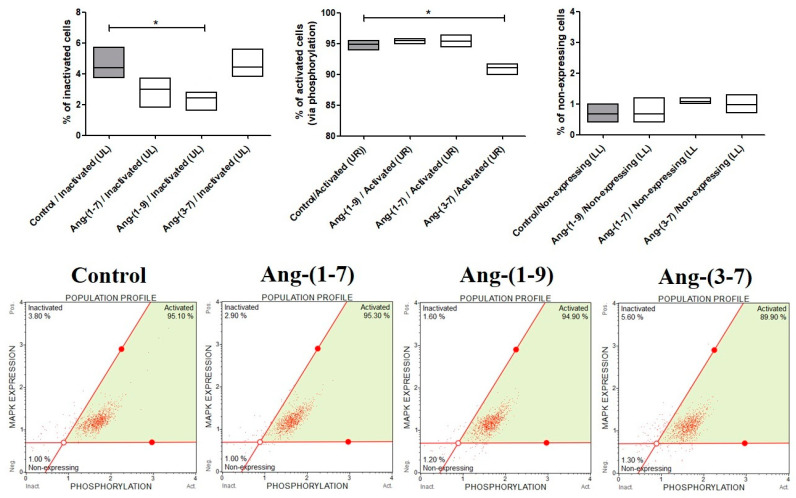
The Muse® MAPK Activation Dual Detection Assay results, following the incubation (48 h) of OVPA8 cells with tested angiotensins in the medium without FBS (mean ± SD; one-way ANOVA with Tukey’s test: * *p* < 0.05).

The effect of angiotensin peptides on MAPK activation was analyzed by flow cytometry assessing the expression of phosphorylated ERK1/2 (Thr202/Tyr204, Thr185/Tyr187) in OVPA8 cells. The percentage of inactivated cells decreased after 48 h incubation with 0.1 nM Ang-(1-7) and Ang-(1-9) but significantly only for the last one. Ang-(3-7) caused a slight change but significantly decreased the percentage of activated ERK1/2 in ovarian cancer cells (Figure 5).

### 2.4. The Changes in the mRNA Expression of NFkB Family Members in OVPA8 Cells after Angiotensin Peptides Exposure

The NF-κB signaling pathway plays an important role in regulating the survival and metastasis of ovarian cancer cells. As shown in Table 1, all angiotensins increased gene expression of *RELB* in OVPA8 cells, but the results were significant only for Ang-(1-7). Furthermore, we also observed downregulation of *NFKB1* in OVPA8 cells treated with this heptapeptide. What is interesting is that the ratio of *NFKB1* to *NFKB2* expression levels significantly decreased in high-grade serous ovarian cancer cells after incubation with all tested peptides. The other genes of the NFkB family, such as *RELA* and *REL*, remained unchanged in all study groups.

### 2.5. The Changes in the Level of Angiotensin Receptors after Angiotensin Peptides Exposure

**Figure 6:**
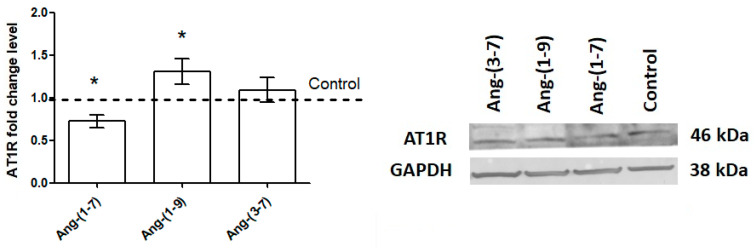
Western blot showing AT1R protein expression after incubation (48 h) of OVPA8 cells with tested angiotensins in the medium without FBS (mean ± SEM; one-way ANOVA with Tukey’s test: * *p* < 0.05).

The OVPA8 cell line expresses angiotensin receptors according to the following trend: *AGTR2* > *AGTR1* ≥ *MAS1* > *AGTR4* (data not shown). The presence of type 1 angiotensin receptor in the OVPA8 cells has also been found at the protein level. The ratio of AT1 to AT2 expression levels significantly decreased after the incubation of OVPA8 cells with Ang-(1-7) and Ang-(3-7). What is interesting is that Ang-(1-7) downregulated the expression of AGTR1 and MAS1 genes while and Ang-(3-7) upregulated the expression of AGTR2 and MAS1 genes. The mRNA level of *AGTR4*/remained unchanged in each case (Table 1). Western blotting largely confirmed the results for ATR1 obtained by RT-qPCR (Figure 6).

## 3. Discussion

Many established cell lines are widely used in ovarian cancer research despite their unclear histological origin. Meanwhile, histological types of ovarian cancer differ not only in origins but also show distinct biology, characteristic molecular profile as well as having an unlikely course of disease and prognosis. 

The OVPA8 cell line is a newly established, well-defined and quite well-characterized model of high-grade serous ovarian cancer (papillary serous adenocarcinoma, G3, FIGO IIIC). This line presents morphologic and genetic features consistent with HGSOC, such as epithelial morphology, multiple chromosomal aberrations, *TP53* and *BRCA1* mutation or loss of one copy of *BRCA2*. Bearing in mind the unequivocally confirmed histological origin of this line, we decided to use it in this study [3].

The presence of all angiotensin receptors on OVPA8 cells may indicate the important role of the renin–angiotensin system in the regulation of biological functions of these cells. The line presented the lower level of mRNA for angiotensin type 1 receptor (*AGTR1*) compared to *AGTR2* gene expression. This may be due to the fact that the crosstalk between *AGTR1* expression and functionality of the *BRCA1* gene has been reported. Real-time PCR and immunohistochemical analysis showed that the levels of AT1R decrease in BRCA1-mutated ovarian cancer [7]. The analysis of three founder mutations of *BRCA1* (C61G, 4153delA and 5382insC) produced negative results in OVPA8 cells, but the homozygous pathogenic mutation c.3700_3704delGTAAA (p.Val1234Glnf) for *BRCA1* gene was identified [3]. We also found the expression of *MAS1* and *AGT4R* encoding a receptor protein for Ang-(1-7) and Ang IV, respectively. 

Few studies on the involvement of the RAS system in the ovarian carcinogenesis process focus primarily on the Ang II/AT1 axis. However, it should be emphasized that Ang II is not the only active peptide of this system, similarly to how AT1 is not the only functional receptor for angiotensin peptides [8,9,10]. Generally, the expression of AT1 is present in most invasive ovarian carcinomas and is not dependent on a histopathologic subtype. Furthermore, it was found that angiotensin receptors type 1 are localized on the membrane and in the cytoplasm of tumor cells, whereas immunohistochemical staining did not detect AT1R expression in the tumor stroma as well as on the surface epithelium of the normal ovary. Suganuma et al. (2005) showed that the AT1R expression is upregulated with the progression from benign to malignant phenotypes of epithelial ovarian tumors [11]. Zhang et al. (2019) presented that grade 1 and 2 tumor patients with high expressions of *AGTR1* had shorter survival times, while for tumor Grade 3, a high level of *AGTR1* expression did not increase the risk of death [12]. On the other hand, Ino et al. (2006) showed no correlation between the level of AT1R and clinicopathological factors (histological subtype, FIGO stage and histological grade) and proliferation marker PCNA [13]. Nevertheless, the influence of AT1R on tumor angiogenesis in ovarian cancer and poor patient outcome was noted. VEGF expression and microvessel density were significantly higher in strongly AT1-positive OC tissues than in weakly positive or negative ones [11,13]. Moreover, the expression of the *AGTR1* gene is significantly positively correlated with EMT marker gene expression [12]. The level of serum angiotensin-converting enzyme (ACE), which is responsible for cleaves of two amino acids from Ang I to form angiotensin II, is significantly higher in epithelial ovarian cancer (EOC) patients than in a control group. Zhang et al. (2019) presented that Ang II treatment increases the formation of multicellular spheroids (MCS), growth and invasiveness of ovarian cancer cell lines by classic direct activation of the MAPK/ERK pathway and transactivation of the epidermal growth factor receptor (EGFR) as well as alleviates ER stress-induced necrosis via modulation of SCD1 levels [12].

In our study we examined the effect of Ang-(1-7), Ang-(1-9) and Ang-(3-7) on cell proliferation and motility of high grade serous ovarian carcinoma (HGSOC). It is worth reminding at this point that Ang-(1-9) is generated from Ang I by a number of different enzymes and can bind to the angiotensin receptors type 1 and 2. Ang-(1-7) is mainly formed from Ang II and Ang-(1-9) and is a ligand for the G-Protein-Coupled Receptor MAS1 and MrgD. Ang-(3-7) can be generated not only from Ang II but also from Ang-(1-7) and has a high relative affinity for AT4 [10,14,15,16]. We observed that the exposure of OVPA8 cells to angiotensins was associated with a change in the level of mRNA for genes of angiotensin receptors. In the case of Ang-(1-7), we noted decreased mRNA levels of AT1 and MAS1 receptors, whereas Ang-(3-7) increased the expression of *AGTR2* and *MAS1* genes. Nevertheless, the impact of both angiotensin peptides decreased the ratio of *AGT1R*:*AGT2R* in OVPA8 cells. Western blot confirmed a decreased level of AT1R after exposure with Ang-(1-7) and an increased level after exposure to Ang-(1-9) and Ang-(3-7), but not all results were statistically significant. In our earlier studies, it was also found that Ang-(3-7) but not Ang-(1-9) can modulate the expression of AT2 and MAS1 in prostate cancer cells [16]. Similarly, in prostate [17] and ovarian cancer cells, Ang-(1-7) can change mRNA expression level not only for the AT1–7/MAS receptor but also for classic angiotensin receptors such as AT1 and AT2.

In this study, we observed that tested angiotensin peptides at the concentration of 0.1 nM can significantly increase the metabolic activity of OVPA8 cells in the culture medium without FBS. Serum starvation is often referred to as “environmental stress” because it reduces levels of growth factors and results in the limitation of cell survival [18]. It appears that angiotensin peptides can modulate the effects of the lack of serum in the culture medium by activation of cell defense mechanisms such as apoptosis. There was no close relationship between the effect of angiotensin peptides on cell viability and the expression of single anti-apoptotic (*BIRC5* and *BCL2*) and pro-apoptotic (*BAX*) genes. However, the level of mRNA *BIRC5* had an upward trend after the exposure to Ang-(1-7) and Ang-(1-9), and we observed a significant increase in the ratio of *BCL2* to *BAX* expression after incubation with Ang (3-7). What is more is that Ang-(1-7) increased gene expression of *RELB* gene, which provides pro-survival functions across multiple cancer types. A similar trend was observed for the other two angiotensin peptides but was not statistically significant. Without exception, all angiotensin peptides decreased the ratio of *NFKB1* to *NFKB2*. On the other hand, angiotensin peptides intensified serum starvation-induced cell cycle arrest at the G0/G1 phase. In the case of Ang-(3-7), a significant decrease in the number of Ki67 positive cells (Ki67+) and a reduced percentage of activated ERK1/2 in ovarian cancer cells were additionally reported. Sobecki et al. (2017) showed that cell–cycle regulation accounts for variability in Ki67 expression. The maximal Ki67 levels are noted in mitosis, and minimal Ki67 levels were noted in late G1 [19]. Ki67 is continuously degraded during G0 and G1; thus, the arrest of OVPA8 cells at G0/G1 explains the low levels of Ki67-positive cells (Ki67+). It is worth noting that the level of Ki67 during G0 and G1 phases in individual cells is very heterogeneous and depends on how long cells spent in quiescence. Recently it has been also reported that Ki67 is not required for proliferation of mammalian cells in culture [20,21]. ERK1 and ERK2 are key players in the control of cell proliferation. It has already been proven that the inactivation of the ERK1/2 MAP kinase pathway results in the suppression of ovarian cancer cell division [22,23]. Ang-(3-7) causes a slight but significantly decreased percentage of activated ERK1/2 in OVPA8 cells. Our results indicate that Ang-(3-7) (0.1 nM) not only increased the accumulation of cells in the G0/G1 phase but also inhibited OVPA8 cell proliferation in the medium without FBS. On the other hand, Ang-(3-7) at the concentration of 10 nM in the medium with 10% FBS showed opposite properties, which included a lowered G1/G0 cell population in favor of the G2/M fraction. We observed similar effects of this peptide in our earlier study on prostate cancer cells. Ang-(3-7) (1 nM; 10% FBS medium) increased the number of LNCaP cells in the G2/M phase and PC3 cells in the S phase [17]. Zhang et al. (2019) presented that Ang II only slightly increased cell proliferation of monolayer cultures but significantly increased cell proliferation in a 3D spheroid model. Authors also showed that Ang II triggers the ERK1/2 and AKT pathways, and the phosphorylation effect is blocked via losartan, an AGTR1 antagonist [12]. The effect of tested angiotensin peptides in 3D conditions would be worth testing; however, in the case of the culture medium without FBS, it was not possible to obtain spheroids for OVPA8 cells.

Meanwhile, ovarian cancer rather disseminates throughout the peritoneal cavity and not via the blood. Thus, with respect to epithelial–mesenchymal transitions, the formation of multicellular spheroids (MCS) and their act of spreading throughout the peritoneal cavity are linked to metastasis. We did not observe any changes in the expression of VIM gene, which is a molecular marker of epithelial–mesenchymal transitions (EMTs), after incubation with any of tested angiotensin peptides. Likewise, there were no differences between the control group and the test groups with regard to the level of *HIF1A* gene, the major mediator of hypoxia-induced EMT. Nevertheless, we observed significant changes in OVPA8 cells mobility after angiotensin exposure. Generally, OVPA8 cells in serum-free medium were very reluctant to migrate. Interestingly, it seems that Ang-(1-7) shows pro-mobility properties in both of the tests used. This is a rather surprising finding, as this peptide is associated rather with the inhibition of metastasis potential of cancer cells. For example, our earlier results clearly show that Ang1–7 can inhibit the anchorage-independent growth and non-adherent colony formation of prostate cancer cells [17]. The evidence for the pro-metastatic role of Ang-(1-7) in cancer cells is scarce. Zheng et al. (2015) noted that Ang-(1-7) improved migratory and invasive abilities of renal cell carcinoma cells (786-O and Caki-1) mediated via the AKT pathway. This effect was abolished by pretreatment with A779, Mas receptor antagonist [24]. It is worth noting that in that study, as in ours, cells were Ang-(1-7)-incubated in the starving medium (1% FBS). We speculate that restriction of growth factors and nutrients in the culture medium may sensitize OVPA8 cells to Ang-(1-7)-induced mobility. It is known that serum starvation can activate or deactivate certain signaling paths in cancer cells. For example, serum starvation in rat microvascular endothelial cells (RMVECs) suppressed ERK1/2 phosphorylation but enhanced p38MAPK [25]. Further investigative studies are required.

In conclusion, we speculate that the adaptation to starvation in nutrient deprived tumors can be modulated by peptides from the renin–angiotensin system. The influence of angiotensin peptides on cancer cells is highly dependent on the availability of growth factors and nutrients.

## 4. Materials and Methods

### 4.1. Cell Lines and Reagents

The research study was conducted on a new stable ovarian cancer cell line, which was derived from a patient with histologically confirmed HGSOC. The OVPA8 cell line was obtained from Prof. Katarzyna Marta Lisowska from the Center for Translational Research and Molecular Biology of Cancer, Maria Skłodowska-Curie Institute-Oncology Center, Gliwice Branch. Detailed analyses confirm that this line is a good model for studies on high-grade serous ovarian cancer, because it has morphologic, molecular and functional features of HGSOC. OVPA8 cells grow within groups attached to the surface. Compared to other ovarian cancer lines these cells have an average ability to divide (doubling time: 44 h), have relatively low migration potential, and possess intermediate invasion rates in Matrigel [3].

OVPA8 cells were cultured in a humidified atmosphere at 37 °C with 5% CO_2_ in RPMI 1640. In addition, standard supplements were used: 10% heat-inactivated fetal bovine serum (FBS), 1 mM sodium pyruvate, 10 mM HEPES buffer and antibiotics (penicillin 50 U/mL; streptomycin 50 mg/mL; neomycin 100 mg/mL).

Angiotensin peptides were purchased from Bachem (Bubendorf, Switzerland): Ang-(1-7) (no. H-1715), Ang-(1-9) (no. H-5038) and Ang-(3-7) (no. H-6965). Peptides were tested in the medium with and without FBS at two time points: 24 and 48 h. The medium containing the above-mentioned compounds was changed every 24 h, because peptides degraded relatively quickly. Unless otherwise specified, the medium and other culture supplements were purchased from Gibco (Thermo Fisher Scientific, Inc., Waltham, MA, USA).

### 4.2. MTT Assay and Alamar Blue Assay

Changes in the cellular viability of OVPA8 after 48 h incubation with angiotensin peptides, Ang-(1-7), Ang-(1-9) and Ang-(3-7), at concentrations of 0.01–10 nM were determined by metabolic activity. The experiments were performed in the medium with or without FBS. The first method (MTT Assay) was based on a reduction in yellow tetrazolium salt 3-(4, 5-dimethylthiazol-2-yl)-2, 5-diphenyltetrazolium bromide by living cells to insoluble purple formazan. The formazan product was dissolved in dimethyl sulfoxide (DMSO). The second method (Alamar Blue Assay) was based on a reduction in blue resazurin by living cells into a water-soluble pink resorufin. The absorbance of the medium was measured by using a microplate reader (BioTek Instruments, Inc., Winooski, VT, USA) at wavelengths of 570 nm and 570/600 nm, respectively. Cell viability (% of control) was calculated in relation to untreated cells.

### 4.3. Assay Muse^®^ Cell Cycle Assay

Changes in the percentage rates of cells in the particular phases of the cycle, G0/G1, S and G2/M, were examined by using a Muse™ Cell Cycle Kit (Luminex Corporation; Austin, TX, USA) according to the manufacturer’s instructions. The experiments were performed in the medium with or without FBS. The ovarian cancer cells after 48 h incubation with angiotensin peptides, Ang-(1-7), Ang-(1-9) and Ang-(3-7), at the concentration of 0.1 nM or 10 nM were trypsinized and fixed in 70% ice-cold ethanol. Fluorescence intensity was measured using a compact cytometry instrument Guava^®^ Muse^®^ Cell Analyzer from Luminex Corporation (Luminex Corporation; Austin, TX, USA). The results are expressed as the percentage of gated cells within each phase. Representative histograms are also shown.

### 4.4. Muse^®^ Ki67 Proliferation Assay

The changes in the proliferation of OVPA8 cells after 48 h incubation with angiotensin, Ang-(1-7), Ang-(1-9) or Ang-(3-7), at the concentration of 0.1 nM were determined by using the Muse^®^ Ki67 Proliferation Kit (Luminex Corporation; Austin, TX, USA). Cells were fixed, permeabilized and stained as per the manufacturer’s instructions. The subpopulations of Ki67-positive and Ki67-negative OVPA8 cells (% gated) were analyzed by using a Guava^®^ Muse^®^ Cell Analyzer from Luminex Corporation. Representative histograms are also shown.

### 4.5. Muse^®^ MAPK Activation Dual Detection Assay

The changes in the levels of ERK1/2 phosphorylation in OVP8 cells after the exposure to 0.1 nM angiotensin were examined by using a Muse^®^ MAPK Activation Dual Detection Kit (Luminex Corporation; Austin, TX, USA). The kit contains two kinds of antibodies: The first antibody measures the phospho-ERK levels, while the second measures total ERK proteins. Ovarian cancer cells after 48 h incubation with Ang-(1-7), Ang-(1-9) or Ang-(3-7) in the medium without FBS were fixed, permeabilized and stained, as per the manufacturer’s protocol. The percentage of the ERK and phospho-ERK proteins was analyzed by a Muse Cell Analyzer (Luminex Corporation; Austin, TX, USA). Representative histograms are also shown.

### 4.6. Wound Healing Assay 

The changes in the speed of wound closure after incubation with angiotensins, Ang-(1-7), Ang-(1-9) and Ang-(3-7), at the concentration of 0.1 nM were examined by using Ibidi Culture-Inserts (Ibidi GmbH, Martinsried, Germany). All procedures were performed according to manufacturer’s instructions. The cells were monitored immediately after gap creation (0 h) and then at time points of 24, 48 and 72 h. The areas of the gap surface and its closure were calculated by ImageJ software v. 152a (Wayne Rasband, National Institutes of Health, Bethesda, MD, USA (http://imagej.nih.gov/ij) (accessed on 7 September 2021).The results are expressed as the fold change relative to control probes.

### 4.7. Boyden Chamber Assay 

The changes in the cell migration of OVPA8 after 48 h incubation with angiotensins, Ang-(1-7), Ang-(1-9) and Ang-(3-7), at the concentration of 0.1 nM were examined by using filters with a 0.8 μm pore size polycarbonate membrane (BD Falcon). The procedure of transwell migration assays was performed as described previously. The migrated cells were stained by crystal violet and dissolved by acetic acid. A quantitative evaluation of cell migration was analyzed by measuring the absorbance at a wavelength of 570 nm. Data were expressed in relation to untreated controls and expressed as the percentage difference of angiotensin-treated cells.

### 4.8. RT-qPCR

After 48 h of incubation with 0.1 nM angiotensin peptides, Ang-(1-7), Ang-(1-9) and Ang-(3-7), OVPA8 cells were harvested in order to extract total RNA with the use of a TRIzol reagent. Next, samples were purified with the standard phenol: chloroform method. The concentration of recovered RNA and its purity were determined by BioDrop μLITE (Tamar Laboratory Supplies Ltd., Cambridge, UK). cDNA was synthesized from 5 μg of total RNA using the ImProm-II™ Reverse Transcription System (Promega, Madison, WI, USA), according to the manufacturer’s instructions. A LightCycler 96 (Roche, Basel, Switzerland) was used to perform RT-qPCR reaction with 2 μL of cDNA. Sequences of primers and annealing temperatures have been previously presented [26]. Quantitative data from gene expression experiments were normalized to the expression of two housekeeping genes: H3F3A and RPLPO. Universal Human Reference RNA (Stratagene, San Diego, CA, USA) was used as a calibrator for each reaction. Differences in gene expression were calculated by Relative Expression Software Tool Multiple Con dition Solver beta software v. 2. (REST-MCS; August 2006) (www.gene-quantification.info) (accessed on 7 September 2021).

### 4.9. Western Blot

The OVPA8 cells were incubated for 48 h with Ang-(1-7), Ang-(1-9), Ang-(3-7) at a concentration 0.1 nM in the medium without FBS. Total protein extracts were isolated from cells using the RIPA protein extraction buffer and supplemented with protease and phosphatase inhibitor cocktails and 1 mM PMSF. The detailed procedures for protein isolation and Western blot analysis have been previously described. The primary antibodies anti-AT1 (Santa Cruz Biotechnology Inc.; Dallas, TX, USA; sc-1173) were detected using conjugated peroxidase-labeled secondary antibodies (Sigma-Aldrich, Saint Louis, MO, USA). Loading control anti-GAPDH (Santa Cruz Biotechnology Inc.; Dallas, TX, USA; sc-59540) was detected using conjugated HRP secondary antibody. Proteins were identified by IGMAFAST™ BCIP^®^/NBT (Sigma-Aldrich, Saint Louis, MO, USA) and chemiluminescence (ChemiDoc, Bio-Rad Laboratories, Hercules, CA, USA), respectively. The optical density of the bands was analyzed using ImageJ software v. 152a (Wayne Rasband, National Institutes of Health, Bethesda, MD, USA) (http://rsb.info.nih.gov/ij/) (accessed on 15 November 2021).

### 4.10. Statistical Analysis

The data are presented as mean ± SD of at least three independent experiments. The measurements were subjected to an analysis of variance (One-Way ANOVA) and the Dunnett’s or Tukey’s multiple comparison test using GraphPad Prism 5 (GraphPad Software, La Jolla, CA, USA) (www.graphpad.com) (accessed on 15 November 2021). Values below *p* < 0.05 were considered statistically significant.

## Figures and Tables

**Table 1:** The fold change values for RT-qPCR results (treatment vs. control), following the incubation (48 h) of OVPA8 cells with tested angiotensins in the medium without FBS (mean ± SEM; one-way ANOVA with Dunnett’s test: * *p* < 0.05; ↓—down-regulation gene; ↑—up-regulation gene).

Gene/Protein	Ang-(1-7)	Ang-(1-9)	Ang-(3-7)
*AGTR1* (AT1R)	0.38 (±0.04) ↓ *	1.26 (±0.17)	1.7 (±0.3)
*AGT2R* (AT2R)	0.74 (±0.16)	1.4 (±0.32)	2.1 (±0.58) ↑ *
*MAS1* (MAS1R)	0.65 (±0.08) ↓ *	1.7 (±0.32)	1.9 (±0.33) ↑ *
*AGT4R* (AT4R)	1.00 (±0.07)	1.06 (±0.05)	1.18 (±0.06)
*AGTR1/AGTR2*	0.55 (±0.05) ↓ *	0.98 (±0.05)	0.80 (±0.06) ↓ *
*AR* (AR)	0.64 (±0.04) ↓ *	1.40 (±0.18)	1.16 (±0.12)
*ESR1* (ERα)	1.13 (±0.2)	1.02 (±0.18)	1.11 (±0.20)
*ESR2* (ERβ)	0.84 (±0.16)	2.56 (±0.81)	2.91 (±0.97) ↑ *
*ESR1/ESR2*	1.32 (±0.19)	0.38 (±0.11) ↓ *	0.38 (±0.13) ↓ *
*PGR* (*PRβ)*	0.37 (±0.09)	2.75 (±0.80)	1.43 (±0.58)
*BIRC5* (Survivin)	1.2 (±0.08)	1.2 (±0.06)	1.08 (±0.05)
*BCL2* (Bcl-2)	1.11 (±0.12)	1.8 (±0.43)	3.12 (±0.70) ↑ *
*BAX* (Bax)	1.07 (±0.05)	1.03 (±0.04)	1.15 (±0.06)
*BCL2/BAX*	1.05 (±0.11)	1.7 (±0.43)	2.74 (±0.61) ↑ *
*MKI67* (Ki-67)	1.12 (±0.05)	1.04 (±0.05)	1.07 (±0.04)
*NFKB1* (NF-κB1)	0.85 (±0.06) ↓ *	0.90 (±0.06)	0.92 (±0.05)
*NFKB2* (NF-κB2)	1.19 (±0.03)	1.03 (±0.04)	1.11 (±0.06)
*NFKB1/NFKB2*	0.70 (±0.02) ↓ *	0.86 (±0.02) ↓ *	0.83 (±0.03) ↓ *
*RELA* (RelA)	1.11 (±0.05)	1.06 (±0.01)	1.05 (±0.08)
*RELB* (RelB)	1.65 (±0.06) ↑ *	1.56 (±0.06)	1.57 (±0.15)
*REL* (c-Rel)	0.99 (±0.04)	0.91 (±0.02)	0.93 (±0.04)
*VIM* (Vimetin)	1.2 (±0.16)	0.8 (±0.06)	0.8 (±0.05)
*HIF1A* (Hif1a)	0.99 (±0.12)	0.95 (±0.01)	0.95 (±0.04)

## Data Availability

Most of the data are presented in the study. The data not presented in this study are available upon request from the corresponding author.
